# *In vivo* mapping of the functional regions of the DEAD-box helicase Vasa

**DOI:** 10.1242/bio.201410579

**Published:** 2015-03-20

**Authors:** Mehrnoush Dehghani, Paul Lasko

**Affiliations:** Department of Biology, McGill University, 3649 Promenade Sir William Osler, Montréal, QC H3G 0B1, Canada

**Keywords:** *Drosophila*, RNA helicase, Embryonic patterning, Germ cells, piRNA biogenesis, Pole plasm

## Abstract

The maternally expressed *Drosophila melanogaster* DEAD-box helicase Vasa (Vas) is necessary for many cellular and developmental processes, including specification of primordial germ cells (pole cells), posterior patterning of the embryo, piRNA-mediated repression of transposon-encoded mRNAs, translational activation of *gurken* (*grk*) mRNA, and completion of oogenesis itself. Vas protein accumulates in the perinuclear nuage in nurse cells soon after their specification, and then at stage 10 Vas translocates to the posterior pole plasm of the oocyte. We produced a series of transgenic constructs encoding eGFP-Vas proteins carrying mutations affecting different regions of the protein, and analyzed *in vivo* which Vas functions each could support. We identified novel domains in the N- and C-terminal regions of the protein that are essential for localization, transposon repression, posterior patterning, and pole cell specification. One such functional region, the most C-terminal seven amino acids, is specific to Vas orthologues and is thus critical to distinguishing Vas from other closely related DEAD-box helicases. Surprisingly, we also found that many eGFP-Vas proteins carrying mutations that would be expected to abrogate DEAD-box helicase function localized to the nuage and posterior pole, and retained the capacity to support oogenesis, although they did not function in embryonic patterning, pole cell specification, *grk* activation, or transposon repression. We conclude from these experiments that Vas, a multifunctional protein, uses different domains and different molecular associations to carry out its various cellular and developmental roles.

## Introduction

The maternally-expressed *Drosophila melanogaster* gene *vasa* (*vas*) encodes a DEAD-box RNA binding protein that is required for posterior patterning and germ cell specification (Hay et al., 1988b; Lasko and Ashburner, 1990). Vas orthologues are expressed in the germ cell lineage, and linked to germ line development, in many other animals including mammals (Castrillon et al., 2000; Fujiwara et al., 1994; Komiya et al., 1994; Kuznicki et al., 2000; Lehmann and Nüsslein-Volhard, 1991; Olsen et al., 1997; Yoon et al., 1997). The first *vas* alleles that were studied in *Drosophila* have a recessive maternal-effect lethal phenotype, in that homozygous females produce non-viable embryos that lack primordial germ cells (pole cells) and most posterior segmentation (Schüpbach and Wieschaus, 1986a). Subsequent study of null *vas* alleles such as *vas^PH165^* revealed earlier functions in oogenesis; *vas*-null females produce very few mature eggs, and the eggs that are produced have dorsal appendage defects resulting from a failure to activate *gurken* (*grk*) translation in the oocyte (Styhler et al., 1998; Tomancak et al., 1998). Grk is an epidermal growth factor receptor (Egfr) ligand that in normal development is secreted from the anterodorsal corner of the oocyte, activating Egfr in the adjacent follicle cells and thus specifying dorsal fate (González-Reyes et al., 1995; Roth et al., 1995). Vas interacts with the translation initiation factor eIF5B, which is necessary for ribosomal subunit joining (Carrera et al., 2000). A mutant form of Vas, Vas^Δ617^, that has a greatly reduced ability to interact with eIF5B does not activate translation of *grk* (Johnstone and Lasko, 2004) or of *mei-P26*, the product of a gene involved in differentiation of germ line stem cells whose expression is also dependent on Vas (Liu et al., 2009). These results suggest a model whereby Vas activates translation of target mRNAs through preferential recruitment of this initiation factor (Lasko, 2013).

Other studies, however, have implicated Vas in processes that do not appear to involve regulation of translation. In early oogenesis, Vas accumulates in nurse cells in a structure called the nuage, then at stage 10 it is transferred to the oocyte where it accumulates in the posterior pole plasm (Hay et al., 1988a; Hay et al., 1988b; Lasko and Ashburner, 1990). Vas is also required for the assembly of the nuage (Liang et al., 1994), which is the cytoplasmic site for processing of Piwi-associated RNA (piRNA) precursors (Pek et al., 2012). Vas co-immunoprecipitates with RNA precursors produced from major piRNA clusters, and in cooperation with a nuclear DEAD-box helicase, Uap56, facilitates the transport of piRNA precursors from the nucleus through nuclear pores to the cytoplasmic nuage (Zhang et al., 2012). piRNAs target transposon-encoded mRNAs and thus protect the genome against the deleterious effects of uncontrolled transposition events (Klattenhoff and Theurkauf, 2008; Pek et al., 2012). Vas physically interacts with some components of the piRNA pathway thereby assembling an amplifier complex on the transposon-encoded transcripts and promoting the ping-pong cycle (Xiol et al., 2014). In *vas^PH165^* ovaries, many transposon-encoded mRNAs are overexpressed, consistent with an essential role for Vas in piRNA biogenesis (Zhang et al., 2012). Another recent study links Vas to regulation of mitotic chromosome condensation in the *Drosophila* germ line, and shows that this function can be carried out by Vas^Δ617^, and thus is independent of eIF5B association (Pek and Kai, 2011).

These many Vas functions make it difficult to study each individually, especially in the case of later functions such as pole cell specification that depend upon earlier events such as posterior localization. Thus, mutants affecting only a single Vas-dependent process would be valuable. To identify such mutants, and to carry out a structure-function analysis of Vas, we generated a set of mutant *egfp-vas* transgenes, and examined their activity in both a maternal-effect lethal *vas* (*vas^1^*) background and in a *vas*-null background. The Vas protein is organized into three domains: a central region of approximately 400 amino acids that is conserved in all DEAD-box helicases, a rapidly evolving N-terminal region of approximately 200 amino acids that contains numerous RGG motifs, and a short C-terminal region. In other RNA-binding proteins RGG motifs act as ancillary motifs, which increase affinity to RNA or confer sequence specificity for RNA binding (Burd and Dreyfuss, 1994; Darnell et al., 2001; Lamm et al., 1996). The C-terminal region contains some amino acids which are conserved in Vas orthologues in other *Drosophila* species and terminates with a stretch of highly acidic residues, conserved in Vas orthologues in *Drosophila* and beyond, but not in other DEAD-box helicases.

In this study we generated several *egfp-vas* constructs with deletions in the N-terminal region and one that additionally abrogates remaining RGG motifs. By expressing these eGFP fusion proteins in a *vas^PH165^* or a *vas^1^* background, we tested the significance of the N-terminal sequence, in general, and the RGG motifs, in particular, for localization of Vas and for its different cellular and developmental functions. Similarly, we investigated the importance of a conserved motif near the C-terminus as well as the highly acidic sequence at the C-terminal end. In addition, we examined the effects on Vas function *in vivo* of non-conservative missense mutations in canonical DEAD-box helicase motifs that would be expected to render the protein catalytically inactive.

## Materials and Methods

### Fly stocks and transgenic lines

*vas^PH165^* is a null allele, lacking the entire coding region of *vas*, which was generated by imprecise P-element excision (Styhler et al., 1998). *vas^1^* is a hypomorphic allele in which there is no amino acid substitution in the coding region but Vas expression is limited to the germarium (Lasko and Ashburner, 1990; Liang et al., 1994; Schüpbach and Wieschaus, 1986b). To generate *egfp-vas* constructs, the open reading frame (ORF) of *vas* was first cloned into a pENTR vector (Life Technologies) with *Xho*I and *Not*I restriction sites added to the 5′ and 3′ ends, respectively. This construct was used to generate deletions or point mutations in the *vas* sequence by PCR-based site-directed mutagenesis. The *Xho*I/*Not*I digested *vas* ORF from this construct was then inserted into *P*[*w*+;*Pvas*-*egfp*] (Nakamura et al., 2001). *egfp-vas^+^* and *egfp-vas^Δ617^*, which were previously generated (Johnstone and Lasko, 2004), and all the N-terminally deleted *egfp-vas* constructs were based on a cDNA clone that lacks one copy of a 39 nucleotide tandem repeat, encoding amino acids 141-153 (Lasko and Ashburner, 1988). The *egfp-vas^G294A^*, *egfp-vas^E400A^* and *egfp-vas^D554A^* constructs also lack these 39 nucleotides as well as the sequence encoding amino acids 15-75. P element-mediated germ-line transformation was performed using a standard technique (Rubin and Spradling, 1982).

### Western blots

To compare protein levels between different transgenic lines, ovary lysates from 2–5 day-old females were resolved on 10% SDS-PAGE gels. Proteins were then transferred onto nitrocellulose membranes for immunoblotting. Primary antibodies were anti-Vas (1:10,000), anti-tubulin (1:15,000, Sigma) or anti-GFP (1:2500, Life Technologies). To examine stability of eGFP-Vas in the pole plasm, protein lysates were prepared from 0–2 h old embryos and processed as for the ovary lysates.

### Egg-laying assay

For each experiment four or five females, after eclosion, were paired with the same number of males and allowed to lay eggs on grape juice plates supplied with yeast for 3 days at 25°C. Eggs were collected and counted once per day. The embryos were also scored for dorsal appendage morphology.

### Hatching assay

For this assay, virgin *vas^1^* females, expressing different *egfp-vas* transgenes, were paired with Oregon-R males and allowed to lay eggs on yeasted grape juice plates. Overnight embryo collections were incubated at 25°C for 48 h, and then larvae and unhatched eggs were counted.

### Immunostaining and *in situ* hybridization

Ovaries from 2–4 day old females were dissected in PBS and fixed for 20 min in 200 µl of PBS containing 4% paraformaldehyde and 0.5% NP-40, plus 600 µl heptane. To immunostain embryos they were collected for 5 h and then fixed in 3 ml PBS containing 4% paraformaldehyde, plus 3 ml heptane. The fixed embryos were stored in methanol at −20°C. Immunostaining was performed according to the protocol described by (Liu et al., 2009). Anti-Vas (1:1000) and anti-Grk (1:500) were used as the primary antibodies. Fluorescent *in situ* hybridization on the embryos was carried out according to the protocol described by Lécuyer et al. (Lécuyer et al., 2007). *fushi tarazu* (*ftz*) cDNA was amplified by PCR from total cDNA synthesized from embryonic mRNA, using gene-specific primers with T3 or T7 promoter sequences added to their 5′ ends. This PCR product was then verified by sequencing and used as a template to produce a digoxigenin (DIG)-labeled probe. Horseradish peroxidase-conjugated anti-DIG (Jackson Immunoresearch) and Alexa Fluor 555 tyramide (Life Technologies) were used to detect the probe after hybridization. Samples were examined under a fluorescent Leica DM6000B microscope. Confocal images were captured using a Zeiss LSM510 microscope.

### Reverse transcription quantitative PCR (RT-qPCR)

Total RNA was extracted from ovaries of 1- to 2-day-old females, using TRI reagent (Sigma). Samples were then treated with Turbo DNase (Ambion) and the RNA was precipitated using acid phenol: chloroform (Ambion). cDNA was synthesized from 2 µg RNA using Maxima H Minus First Strand cDNA synthesis kit (Thermo Scientific). Random hexamers were used for reverse transcription, and to enrich *rp49* cDNA a specific RT primer (5′-TTGGAGGAGACGCCG-3′) was added to the mixture. The resulting cDNA was used for RT-qPCR using *HeT-A-1*, *18S* rRNA and pre-*rp49* primers described by (Zhang et al., 2012). RT-qPCR was performed in a CFX96 Real-Time machine (Bio-Rad) using DyNAmo Flash SYBR Green (ThermoScientific). The expression level of *HeT-A* was quantified relative to *18S* rRNA and pre-*rp49*. A minimum of three biological replicates were tested for each genotype. The graph shows the average and the error bars indicate the standard error of the mean (SEM).

### Live imaging

Embryos were collected for 30 min, then dechorionated using 5% bleach for 1 min and rinsed with water. Embryos were then aligned in a Fluorodish cell culture dish (World Precision Instruments) and covered with Halocarbon oil 400. Live imaging was performed using a WaveFX spinning disk confocal system (Quorum) and a DM6000B inverted microscope (Leica). Images were captured and processed using Volocity 3D image analysis software (PerkinElmer).

### Statistical analyses

A two-tailed t-test was used to calculate the p-values in each assay. The difference between two constructs was considered significant if the p-value was less than 0.05. To assess the effect of different deletions or point mutations on Vas function, the mutated *egfp-vas* constructs were compared to the wild type control (*egfp-vas^+^*), unless otherwise specified.

## Results

### A series of eGFP-Vas proteins with mutations in the N-terminus, conserved helicase domains and the short C-terminus

**Fig. 1.**
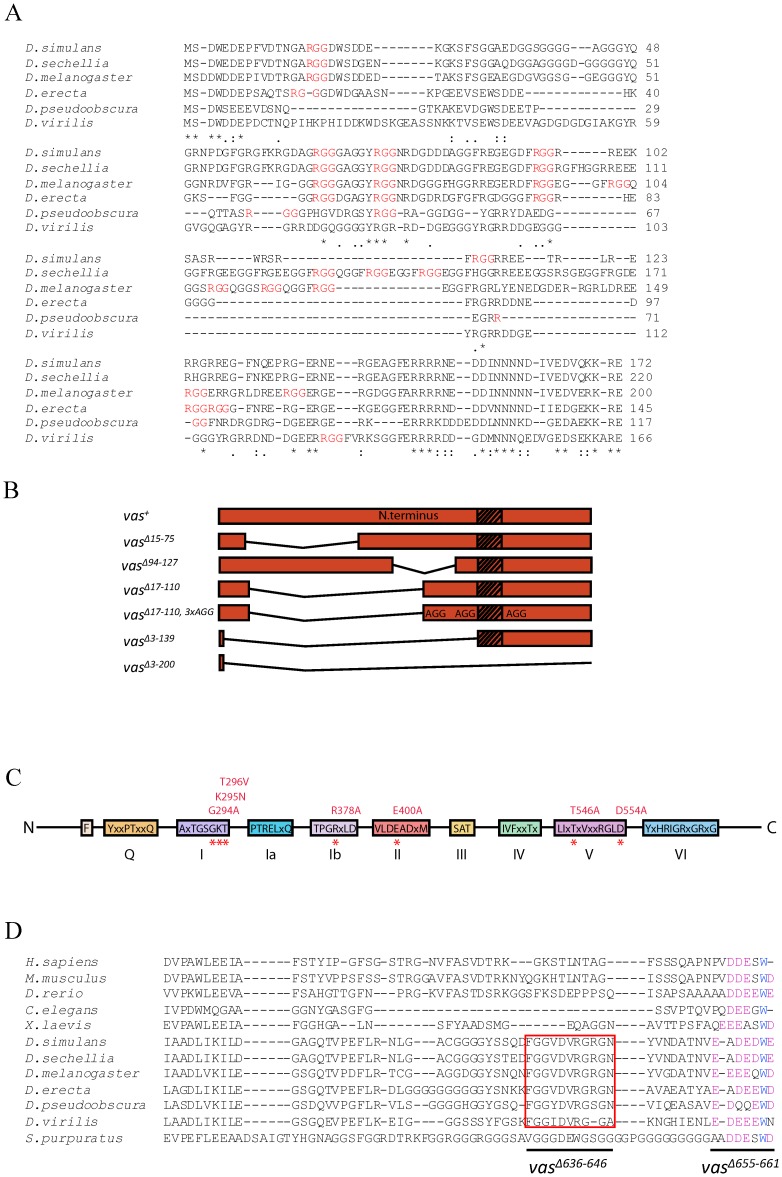
Summary of the deletions and point mutations examined in this study. (A) An alignment of the N-terminal ends of predicted Vas proteins from several *Drosophila* species. The N-terminal *vas* open reading frame is often incorrectly annotated in species that have not undergone extensive cDNA sequencing because of confounding factors such as poor sequence conservation, nested genes and the *solo* alternative splice form (Yan et al., 2010). Therefore, to produce this alignment, *vas* open reading frames were manually annotated from three-frame translations of genomic DNA, using the short highly-conserved amino-terminal end to identify the putative translational start site. Asterisks, colons and periods indicate full conservation, strong similarity and weak similarity, respectively. RGG motifs are shown in red. (B) Schematic representation of the N-terminal deletions used in this study. The hash box marks amino acids 141-153, which are encoded by a copy of a 39-nucleotide tandem repeat that is absent from some *vas* cDNA clones (Lasko and Ashburner, 1988). This segment is absent in eGFP-Vas^+^ and all the N-terminally deleted proteins as the constructs were built from such a cDNA clone. Vas^Δ17-110, 3xAGG^ contains a deletion of amino acids 17-110 and three mutations that convert RGG motifs to AGG. (C) The amino acid substitution mutations in conserved DEAD-box helicase motifs that were produced for this study. Motifs are identified as previously defined (Rocak and Linder, 2004). (D) Sequence alignment of the C-terminal region of Vas from *D. melanogaster* with orthologues from other species. The red box depicts amino acids 636-646, which are conserved among *Drosophila* species but not beyond. The purple letters show the conserved highly acidic residues found at the C-terminal ends of Vas orthologues from *Drosophila* and non-*Drosophila* species. A tryptophan residue (presented in blue) is also highly conserved.

The N-terminal region of Vas evolves rapidly; there are numerous sequence changes even between *D. melanogaster* and the closely related species *D. simulans* (Fig. 1A). Nearly all Vas orthologues contain several RGG repeats within this region, although the number of such motifs and their spacing vary considerably. To examine the role of the variable N-terminal domain in Vas function, we produced *egfp-vas* transgenic constructs deleted for different segments of the N-terminal region (*egfp-vas^Δ15-75^, egfp-vas^Δ17-110^, egfp-vas^Δ94-127^, egfp-vas^Δ3-139^, egfp-vas^Δ3-200^*; Fig. 1B). In addition, to specifically test the role of RGG motifs, we mutated arginine to alanine in the three RGG motifs that remain present in *egfp-vas^Δ17-110^ (egfp-vas^Δ17-110, 3xAGG^)*; the three arginines mutated are R115, R122, and R163.

To examine the role of the conserved DEAD-box domains, we produced *egfp-vas* transgenic constructs with the following point mutations: G294A, K295N, T296V, R378A, E400A, T546A, and D554A (Fig. 1C). Nine highly conserved motifs have been identified in DEAD-box helicases (Rocak and Linder, 2004). The first three of these mutations affect motif I, which is the Walker A motif, a well-established ATP binding domain conserved throughout evolution (Cordin et al., 2006; Walker et al., 1982). R378A affects motif Ib; E400A affects motif II, the DEAD-box; and the final two mutations affect motif V.

Near the C-terminal end, Vas contains a region (amino acids 636-646) that is conserved among closely related *Drosophila* species and that includes a potential arginine methylation site (R644). The C-terminal sequence also ends with a highly acidic region (amino acids 655-661), which is found in many Vas orthologues. To examine the functional significance of these sequences in the C-terminal regions we generated *egfp-vas^Δ636-646^*, *egfp-vas^R644A^* and *egfp-vas^Δ655-661^* transgenic lines (Fig. 1D).

The *egfp-vas^+^* wild-type control and all the constructs with N-terminal deletions were generated from a *vas* cDNA that lacks one copy of a 39 nucleotide tandem repeat, encoding amino acids 141-153 (Lasko and Ashburner, 1988). The G294A, E400A and D554A constructs also carried the Δ15-75 deletion. Neither the deletion of amino acids 15-75 nor 141-153 has any measurable effect on Vas localization or function (Lasko and Ashburner 1990; Styhler et al. 1998; this work). All the other constructs were generated from a full-length *vas* cDNA.

### Most non-null mutations of *vas*, including some affecting residues highly conserved in DEAD-box helicases, can support egg production

**Fig. 2.**
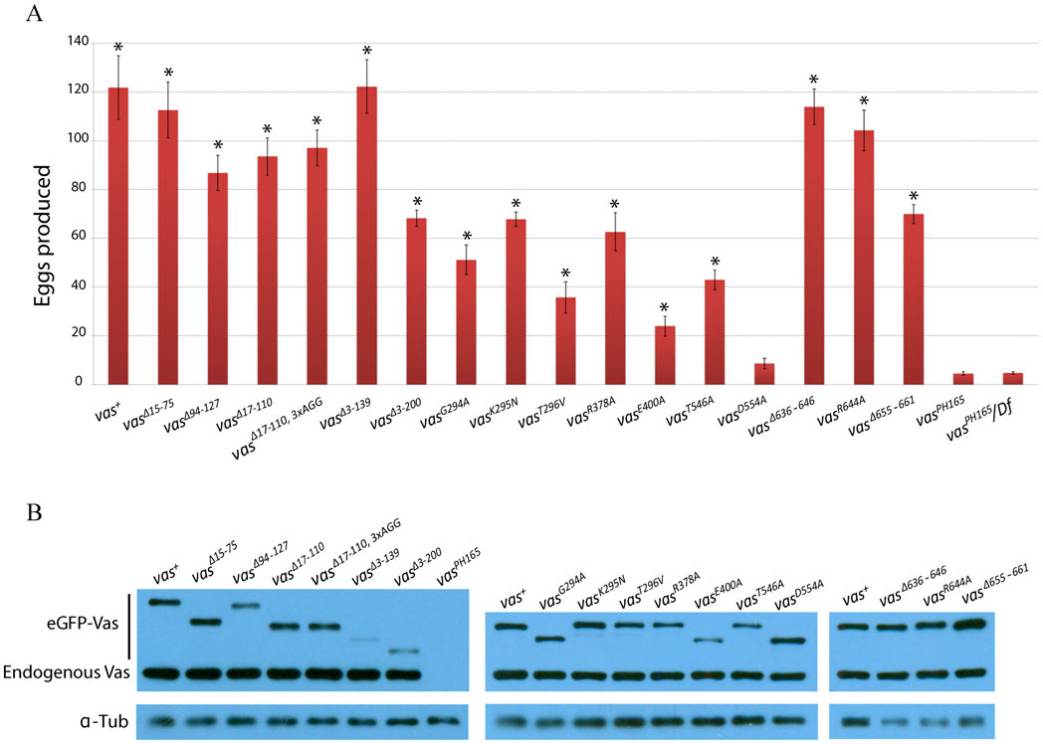
Fecundity of *vas^PH165^* females carrying different *egfp-vas* constructs, and expression levels from those constructs. (A) Fecundity. The y-axis indicates the number of eggs laid by individual females in the first three days after eclosion, and the x-axis identifies each *egfp-vas* construct that was tested. Data from *vas^PH165^* and *vas^PH165^/Df(2L)A267* controls are presented at the far right. Asterisks indicate a significant increase compared to *vas^PH165^* (p<0.05). Error bars indicate the standard error of the mean (SEM), n (number of females tested) >50 for all genotypes. (B) Western blots (WB) comparing the expression level of eGFP-Vas in the ovaries from *vas^1^*/+ flies carrying the different constructs, using an anti-Vas antibody. α-Tubulin (α-Tub) serves as a loading control. *egfp-vas^+^* was included in each blot for comparison. The eGFP-Vas bands migrate at different positions depending on the deletions that they carry. No Vasa protein was detected in the ovary lysate from *vas^PH165^*. The Vasa antibody raised against the full-length protein reacts with some mutant forms of Vasa such as *vas^Δ3-139^* and *vas^Δ3-200^* less strongly than with *vas^+^* (see also supplementary material Fig. S1).

Females homozygous for *vas* deletions produce very few mature eggs (Styhler et al., 1998). To examine which domains of Vas are essential for oogenesis we expressed eGFP-Vas proteins with various mutations in a *vas^PH165^* mutant background, and counted the number of eggs produced (Fig. 2A). For these and subsequent experiments, we selected individual transgenic lines that gave comparable to or higher levels of eGFP-Vas expression than the *egfp-vas^+^* wild-type control (Fig. 2B; supplementary material Fig. S1).

In this manner, we found that deletion of the first 200 N-terminal amino acids, or of smaller segments within the N-terminal region, had less than a two-fold effect on female fecundity (Fig. 2A). This indicates that sequences in the N-terminal region of Vas are largely dispensable for completion of oogenesis. Quite surprisingly, we also only observed modest effects (2–3 fold reduced from the *egfp-vas^+^* wild-type control) on egg production from females that expressed eGFP-Vas bearing one of three non-conservative mutations in the Walker A motif (G294A, K295N, T296V) or either of two mutations (R378A and T546A) in other highly conserved DEAD-box helicase motifs (Fig. 2A). A slightly larger effect (around 5-fold) was observed from E400A. All these mutant forms of eGFP-Vas rescued egg production to *vas^PH165^* mutant females at a statistically significant level (supplementary material Table S1). The Walker A and B motifs comprise a very well characterized and widespread ATP-binding domain (Cordin et al., 2006; Walker et al., 1982), and the four non-conservative substitutions in residues 294-296 and 400 would be expected to abrogate its function. R378A impacts DEAD-box helicase motif Ib, which in eIF4A is essential for RNA binding (Rogers et al., 2002), while T546A affects motif V, which is shown to couple the ATPase and helicase activities in other DEAD-box helicases (Caruthers et al., 2000; Sengoku et al., 2006). Taken together, these results indicate that mutant forms of Vas with non-conservative amino acid substitutions in motifs implicated in ATP binding, and thus also in its RNA helicase activity that depends on ATP hydrolysis, can nevertheless rescue oogenesis to *vas*-null females to a substantial degree. The sole point mutation that did not restore fecundity to *vas^PH165^* females was D554A, which affects a highly conserved residue of motif V.

We also examined mutants affecting C-terminal residues well conserved among Vas orthologues but not among other DEAD-box helicases for their effects on oogenesis. Similar to the N-terminal deletions, these mutant forms of eGFP-Vas were able to restore fecundity to *vas^PH165^* females 50–100% as effectively as a wild-type eGFP-Vas fusion (Fig. 2A).

### Vas requires the conserved DEAD-box domains as well as the acidic C-terminus for the translational activation of Grk

**Fig. 3.**
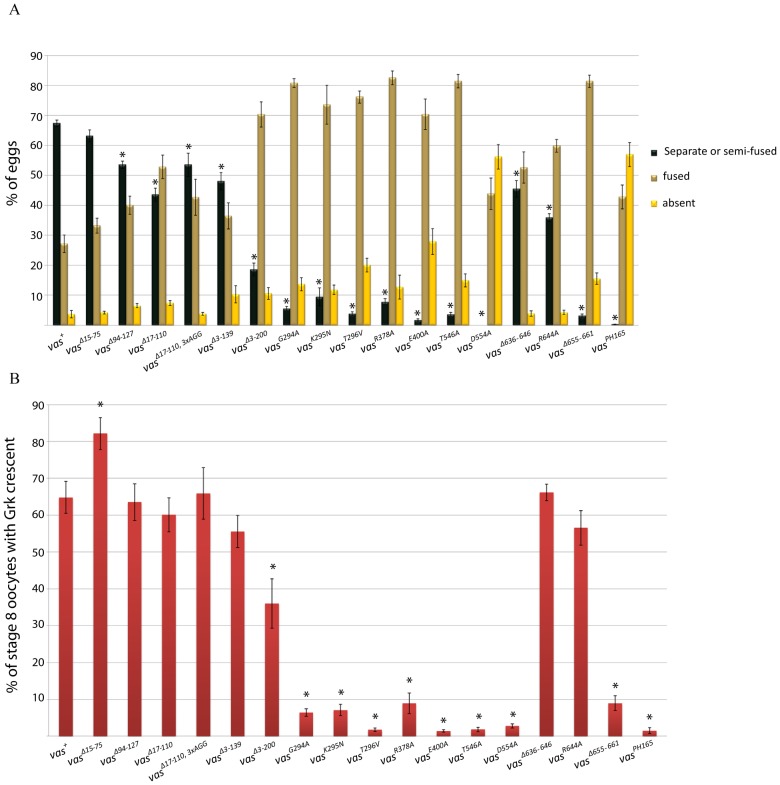
Dorsal-ventral patterning in eggs produced by *vas^PH165^* females carrying different *egfp-vas* constructs. (A) Dorsal appendage formation. The green bars indicate the percentage of the embryos with two separate or partly fused dorsal appendages. The beige and yellow bars represent the portion of the embryos with fused or no dorsal appendages, respectively. Data from *vas^PH165^* controls are presented at the far right. Error bars indicate SEM, n (number of females tested) >50 for all genotypes. Asterisks indicate a significant difference compared to *vas^+^* (p<0.05). (B) Grk expression. Red bars indicate the percentage of stage 8 egg chambers positively stained for localized Grk. Error bars represent SEM from three different replicates. n (number of stage 8 egg chambers in each replicate) >50 for all genotypes.

Females null for *vas* function fail to translate *grk* mRNA at wild-type levels (Styhler et al., 1998; Tomancak et al., 1998), and the consequent reduction of secreted Grk ligand available to adjacent follicle cells results in ventralization of their patterning, which can be observed through effects on the structure and position of the dorsal appendages of the eggshell. As patterning becomes more ventralized, the dorsal appendages move closer together, then fuse, and then become absent altogether. We compared the percentages of eggs produced from each line expressing a different eGFP-Vas mutant in a *vas^PH165^* mutant background that had two dorsal appendages (separate or partly fused), a single fully fused appendage, or no appendages, as an index of Gurken (Grk) expression (Fig. 3A). Using this assay, we found that most N-terminal deletions of eGFP-Vas had only modest, but nevertheless statistically significant, effects on dorsal appendage formation (Fig. 3A; supplementary material Table S1). Larger N-terminal deletions had somewhat larger effects with greater statistical significance, and fewer than 20% of eggs produced from females expressing only eGFP-Vas deleted for amino acids 3-200 had two dorsal appendages (Fig. 3A; supplementary material Table S1). In contrast to what we observed for oogenesis, all mutations in the conserved helicase domains severely compromised dorsal appendage formation (less than 10% of embryos with two dorsal appendages; Fig. 3A), and again D554A had the most extreme effect (no embryos with two appendages). The three C-terminal mutations we examined behaved divergently in this assay; deletion of amino acids 636-646 or a point mutation in a conserved residue within this segment (R644A) had only modest but statistically significant effects, while deletion of the seven most C-terminal amino acids (655-661) produced nearly as severe a phenotype as some of the mutations in conserved DEAD-box domains (Fig. 3A; supplementary material Table S1).

We also examined Grk expression directly in these mutants by immunohistochemical staining of stage 8 egg chambers, where in wild-type a prominent crescent of Grk is apparent adjacent to the nucleus in the anterodorsal corner of the oocyte. We counted the percentage of stage 8 oocytes with a visible Grk crescent, and obtained results that were consistent with those we observed for dorsal appendage formation (Fig. 3B). Again, mutations affecting conserved DEAD-box helicase residues, and deletion of amino acids 655-661, showed strong effects (>85% decrease in the number of stage 8 egg chambers with a Grk crescent compared to *vas^+^*). The other constructs could largely rescue Grk expression in *vas^PH165^* oocytes with the exception of *egfp-vas^Δ3-200^* where a Grk crescent was visible in only 36±6.6% of the oocytes (p = 0.029 when compared to *egfp-vas^+^*). The Grk crescent was typically less pronounced in mutants where it was visible in a smaller proportion of oocytes (supplementary material Fig. S2). Both the indirect dorsal appendage assay and the direct immunohistochemical assay indicated that Vas requires its conserved DEAD-box helicase motifs, as well as its seven most extreme C-terminal amino acids, for translational activation of Grk. This is consistent with our earlier hypothesis that Vas actively promotes *grk* translation (Johnstone and Lasko, 2004).

### The N-terminal and C-terminal domains as well as the conserved core region contain important elements for Vas localization

Next, we analyzed our collection of *egfp-vas* mutants in a *vas^1^* genetic background to assess phenotypes that manifest themselves at later developmental stages. Expression of Vas in the *vas^1^* mutant is restricted to the germarium stage, and this mutant produces wild-type numbers of eggs, which however do not support germ cell specification or posterior patterning (Hay et al., 1990; Lasko and Ashburner, 1990; Schüpbach and Wieschaus, 1986a). For simplicity we will refer to embryos produced by *vas^1^* mothers as “*vas^1^* embryos”.

**Fig. 4.**
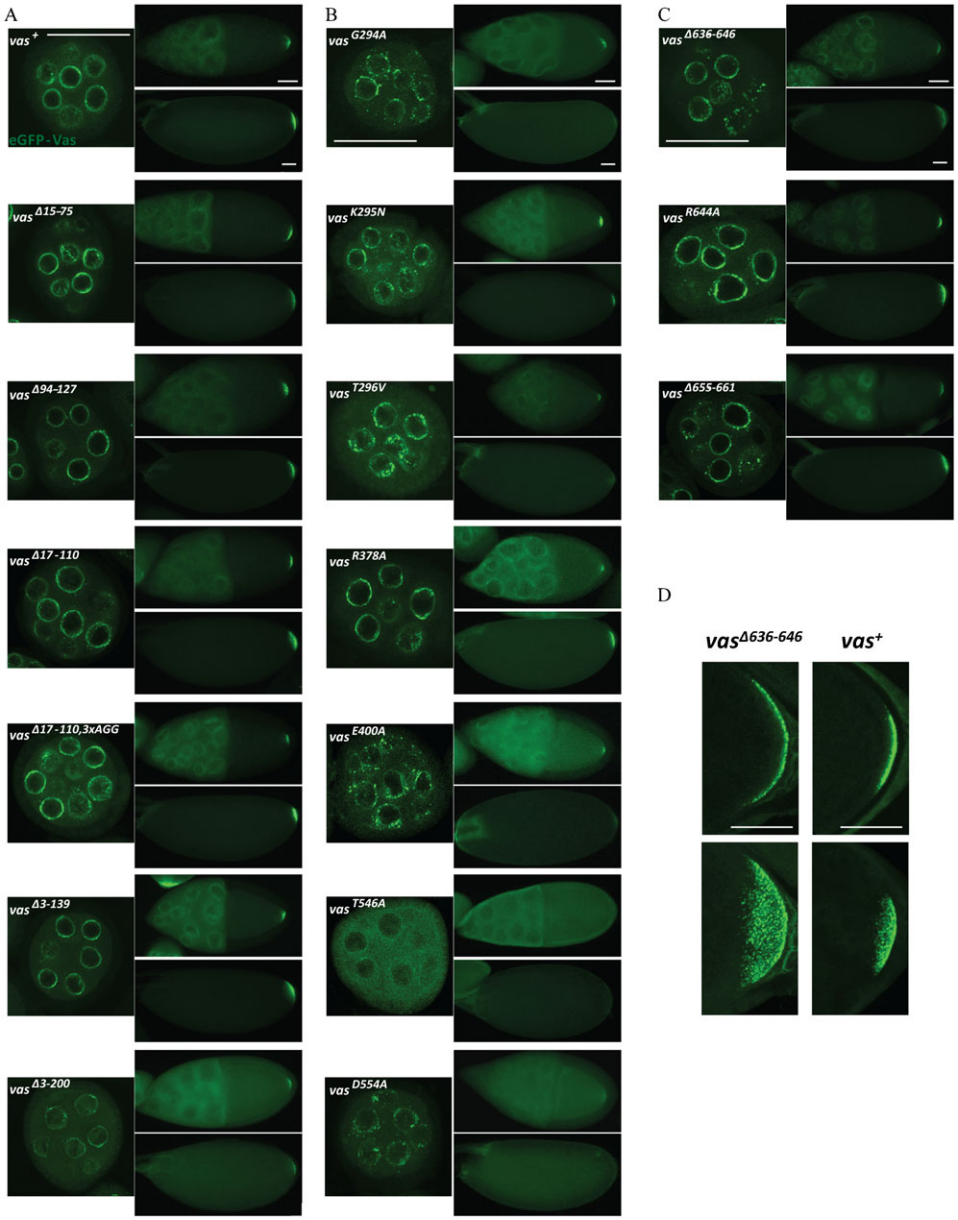
Localization of eGFP-Vas proteins in *vas^1^* ovaries. (A) N-terminal deletions. (B) Mutations in conserved DEAD-box helicase motifs. (C) C-terminal mutations. For each genotype the left, the top right and the bottom right images show a stage 5 egg chamber (confocal image), an early stage 10 egg chamber, and a stage 14 oocyte. Scale bars (50 µm) are included on the top set of images; all corresponding images from other genotypes are at the same magnification. (D) Higher magnification confocal images comparing distribution of eGFP-Vas at the posterior pole of *vas^1^; egfp-vas^+^* and *vas^1^;egfp-vas^Δ636-646^* stage 14 oocytes. eGFP-Vas^Δ636-646^ distribution is more diffuse. For each genotype the top image shows the middle focal plane whereas the bottom image illustrates the maximum intensity projection of the z stack. Scale bars = 50 µm.

First, we examined the spatial distribution of each mutant form of eGFP-Vas. In early oogenesis, wild-type Vas accumulates in the perinuclear nuage of the nurse cells. Beginning at stage 10, Vas is then transported into the oocyte where it accumulates in the posterior pole plasm. This localization pattern is recapitulated by transgenically expressed wild-type eGFP-Vas, and by eGFP-Vas deleted for amino acids 3-139 or for several smaller segments within that interval (Fig. 4A). However, a more extensive N-terminal deletion of amino acids 3-200 made the association of Vas with the perinuclear nuage weaker and reduced Vas localization to the pole plasm (Fig. 4A).

Mutations affecting conserved DEAD-box residues had different effects on Vas localization. Surprisingly, most of the eGFP-Vas mutants we analyzed retained some ability to localize to the nuage in stage 5 nurse cells and to the posterior pole of the stage 10 oocyte (Fig. 4B). The single exception was T546A, which presented an almost completely uniform distribution. For most other mutations affecting conserved DEAD-box residues, nuage-associated eGFP-Vas was distributed in a far more punctate pattern than in wild-type or in all N-terminal deletions except for Δ3-200 (compare Fig. 4B with Fig. 4A). Posterior localization of most mutant eGFP-Vas proteins affecting conserved DEAD-box residues was transient and did not persist through the end of oogenesis. The R378A allele is an exception in that nuage localization appeared relatively normal and posterior localization was stable, persisting into early embryogenesis (Fig. 4B). To a lesser extent, localized eGFP-Vas was also apparent in embryos produced from *vas^K295N^* mothers (Fig. 4B).

None of the C-terminal mutants we analyzed had drastic effects on localization; however, posterior accumulation of eGFP-Vas deleted for amino acids 636-646 was less robust and more diffuse than in wild-type (Fig. 4C,D).

### The role of Vas in repressing transposon-encoded mRNAs requires its helicase function and is also dependent on some N-terminal and C-terminal motifs

**Fig. 5.**
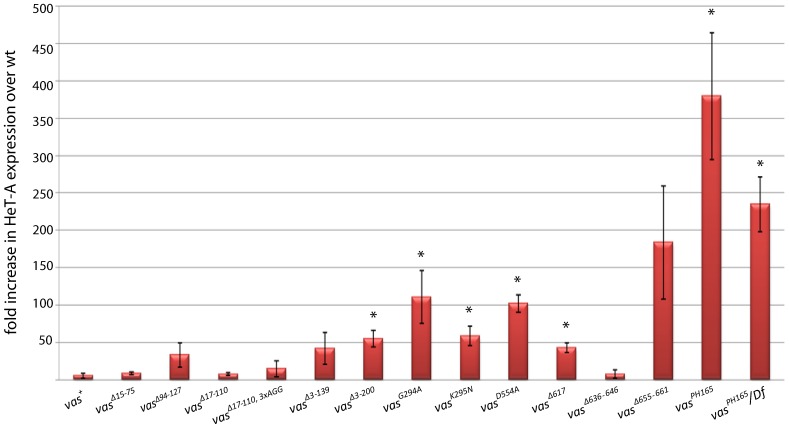
HeT-A expression in ovaries of *vas^PH165^* females carrying different *egfp-vas* constructs. Red bars indicate the expression level of HeT-A normalized to that of wild-type ovaries. Data from *vas^PH165^* and *vas^PH165^/Df(2L)A267* controls are presented at the far right. Asterisks indicate a significant increase compared to *vas^+^* (p<0.05). Each bar represents the average of at least three biological replicates, error bars indicate SEM.

Females lacking *vas* function overexpress mRNA from a subset of transposon families in their ovaries (Zhang et al., 2012). We examined levels of HeT-A, a retrotransposon that is highly expressed in *vas* deficient ovaries, in *vas^PH165^* females carrying different *egfp-vas* constructs. Consistent with a previous report (Zhang et al. 2012), quantitative RT-PCR (RT-qPCR) analyses indicated that the expression level of HeT-A in the ovaries of homozygous *vas^PH165^* or *vas^PH165^*/*Df(2L)A267* females is more than 100-fold higher than in wild-type controls (Fig. 5). eGFP-Vas^+^, as well as the N-terminally truncated proteins lacking the interval 17-110 or shorter, could suppress HeT-A expression in the *vas^PH165^* ovaries to within an order of magnitude of the wild-type level. On the other hand, expression of HeT-A in ovaries expressing the largest N-terminal deletion (eGFP-Vas^Δ3-200^) was about 50-fold higher than wild-type (p = 0.0039), indicating some requirement for N-terminal sequences between amino acids 111-200 in repression of transposon-encoded gene expression. HeT-A expression in *vas*-null ovaries expressing constructs that delete part of this region (*egfp-vas^Δ3-139^* or *egfp-vas^Δ94-127^*) was highly variable from experiment to experiment, ranging from near wild-type levels to levels similar to *vas ^Δ3-200^*, and because of this variability the difference from *egfp-vas^+^* was not statistically significant (supplementary material Table S1). We observed that HeT-A expression in *vas^PH165^*; *egfp-vas^Δ17-110, 3xAGG^* ovaries was also highly variable (Fig. 5).

Overexpression of HeT-A by 50- to 100-fold was found when ovaries expressing any of three mutant forms of eGFP-Vas affecting conserved DEAD-box domains (G294A, K295N, or D554A) were examined (p<0.05 in all three cases). Surprisingly, Vas^Δ617^, a mutant that reduces the interaction with eIF5B and thus affects the role of Vas in translational activation (Johnstone and Lasko, 2004; Liu et al., 2009), also could not rescue suppression of HeT-A expression (p = 0.0024). We further found that deletion of the interval 655-661, covering the highly conserved C-terminal acidic residues, reduces the ability of eGFP-Vas to support HeT-A silencing. While all individual data points for this construct showed elevated HeT-A expression (32-261 fold over wild-type), this high degree of variability rendered this difference not statistically significant (supplementary material Table S1).

### In addition to its core helicase domain, Vas requires motifs in the N-terminus and C-terminus regions to support germ cell specification

**Fig. 6.**
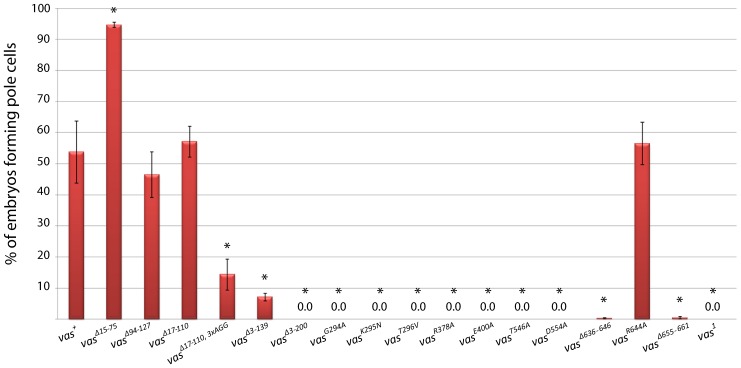
Germ cell formation in embryos from *vas^1^* females expressing different eGFP-Vas proteins. Red bars indicate the percentage of the embryos with germ cells. Data from the *vas^1^* control is presented at the far right. Asterisks show a significant difference from *vas^+^* (p<0.05). Error bars represent SEM from at least three biological replicates each with more than 50 embryos.

eGFP-Vas deleted for amino acids 15-75, 17-110, or 94-127 retained the ability to induce germ cell formation in *vas^1^* embryos to a similar or even greater extent to eGFP-Vas^+^ (Fig. 6). However, deletion of amino acids 3-139 clearly impacted the ability of eGFP-Vas to restore pole cell formation to *vas^1^* embryos (p = 0.018 when compared with eGFP-Vas^+^), with only 7±1.1% of embryos from females expressing this construct forming pole cells. This was not due to low expression level from this construct (supplementary material Fig. S1), but we did find that this protein is unstable in embryos (supplementary material Fig. S3). Next, we examined embryos produced from *vas^1^* females expressing eGFP-Vas^Δ17-110, 3xAGG^ (Fig. 6; supplementary material Fig. S3). In this line the frequency of embryos forming germ cells was only 14±4.9% (p = 0.022 when compared with eGFP-Vas^+^), suggesting that the RGG motifs in the N-terminal region of Vas play a role in germ cell formation. Importantly, all of these forms of eGFP-Vas localize normally to the posterior pole plasm (Fig. 4A). *vas^1^* embryos expressing eGFP-Vas^Δ3-200^ never produced germ cells, further implicating N-terminal motifs in pole cell specification.

Despite the ability of some to localize to the pole plasm, all mutant forms of eGFP-Vas that affect conserved DEAD-box residues completely failed to restore the ability to form pole cells to *vas^1^* embryos. This implies that Vas must be catalytically active to mediate germ cell specification.

**Fig. 7.**
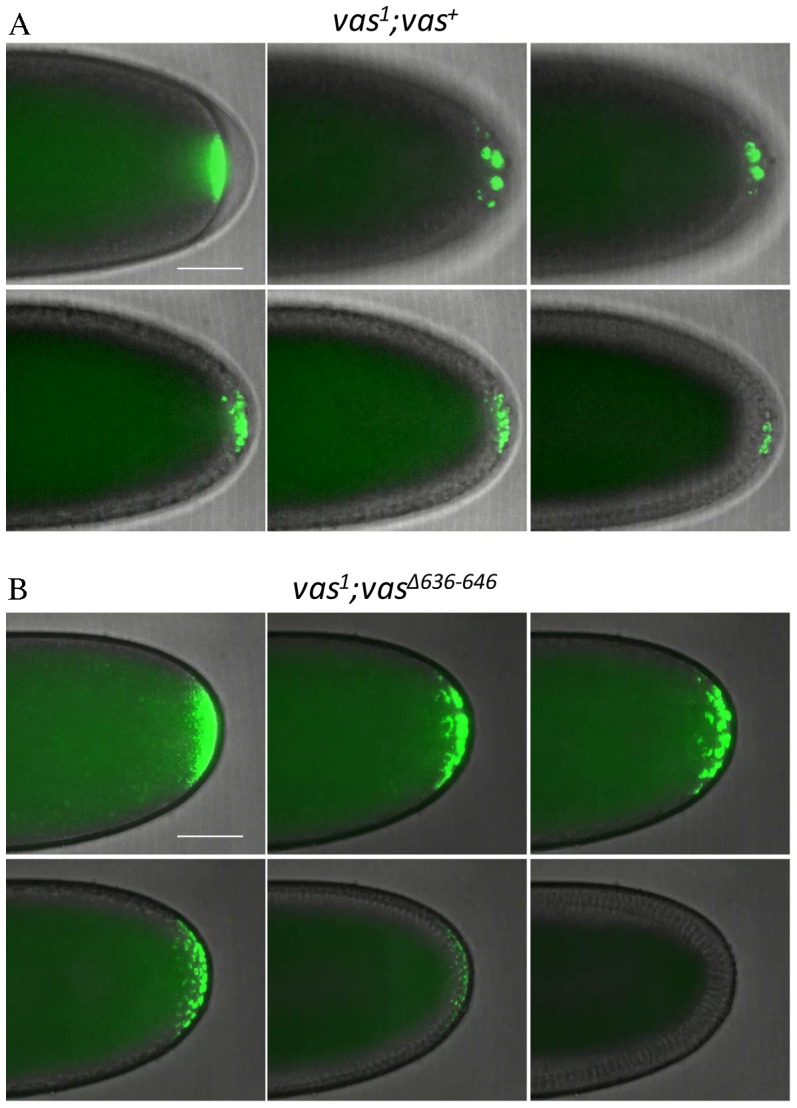
Time course of pole cell development in eGFP-Vas^+^ and eGFP-Vas^Δ636-646^ expressing embryos. (A) A series of still shots from live imaging of pole cell formation in eGFP-Vas^+^ expressing embryos. eGFP-Vas is tightly localized at the posterior pole and then concentrates in foci within pole buds. Posterior nuclear divisions become asynchronous with somatic nuclear divisions, and eGFP-Vas positive pole cells completely form. (B) A series of still shots from live imaging of pole cell formation in eGFP-Vas^Δ636-646^ expressing embryos. eGFP-Vas is less tightly localized at the posterior pole and forms more foci than in wild type. Mitosis remains synchronous throughout the embryo. eGFP-Vas is then lost from the posterior region and pole cells fail to form. Scale bar = 50 µm.

One C-terminal mutant, eGFP-Vas^Δ636-646^, when expressed at levels comparable to the wild-type control, restored almost no pole cell formation activity to *vas^1^* embryos (Fig. 6). When examined in more detail, however, its phenotype is novel in that pole buds begin to form but then regress. Unlike in wild-type, mitotic divisions remain synchronous in the somatic and posterior pole region of embryos expressing eGFP-Vas^Δ636-646^ (Fig. 7; supplementary material Movies 1, 2). We hypothesize that the more diffuse localization of eGFP-Vas^Δ636-646^ at the posterior region results in an insufficient concentration of Vas at the extreme posterior to catalyze pole cell formation, and as mitosis remains synchronous, the eGFP-Vas that is present gets divided into a larger number of foci than in wild-type. Supporting this hypothesis, higher expression of eGFP-Vas^Δ636-646^ enables many more *vas^1^* embryos to produce pole cells, however the number of pole cells each embryo produces is highly variable, and on average is much lower than that for example in *vas^1^; egfp-vas^Δ15-75^*, which is expressed at a comparable level (4.3±0.3 versus 9.9±0.2) (supplementary material Fig. S4). We did not observe a similar phenotype for eGFP-Vas^R644A^, a point mutation that affects a potential arginine methylation site within the 636-646 interval.

Lastly we found that the conserved sequence at the C-terminus of Vasa is required for germ cell formation (p = 0.013; Fig. 6). *vas^1^; eGFP-vas^Δ655-661^* embryos produce almost no germ cells despite the normal localization of eGFP-Vas to the posterior pole.

### Posterior patterning activity of Vas is abolished by removing the entire N-terminal domain or by point mutations in conserved DEAD-box helicase domains

**Fig. 8.**
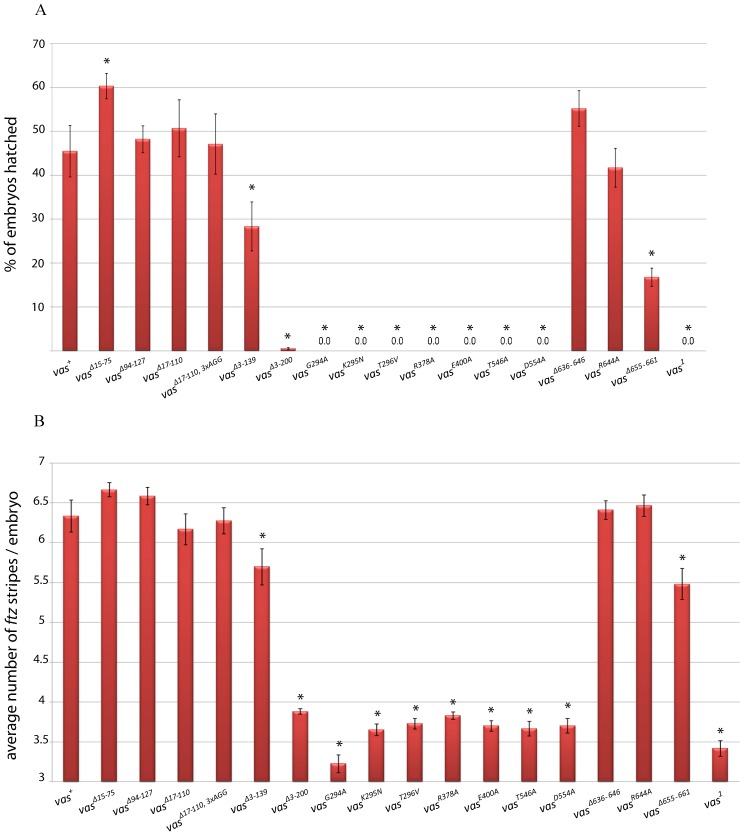
The ability of various *egfp-vas* constructs to restore abdominal segmentation in *vas^1^* embryos. (A) Hatching rates: the red bars indicate the percentage of embryos hatched after 48 hours, error bars indicate SEM from at least five plates. Between 500-1000 embryos in total were scored for each genotype. (B) *ftz* expression in *vas^1^* embryos containing various *egfp-vas* constructs: The y-axis indicates the average number of *ftz* stripes for each genotype. Error bars show SEM calculated from more than 50 embryos examined for each genotype. In both A and B asterisks show a significant increase or decrease from vas^+^ (p<0.05).

*vas^1^* embryos fail to hatch because they lack posterior segments and thus die. We tested the ability of the various eGFP-Vas constructs to rescue embryonic viability to *vas^1^* embryos. Proteins with N-terminal deletions within the interval 15-127 differ little from wild-type eGFP-Vas in their ability to restore viability to *vas^1^* embryos (Fig. 8A). However, embryonic viability decreased about 37% by removing amino acids 3-139 (p = 0.044) and was almost completely abolished by a more extensive N-terminal deletion (Δ3-200). We also found that all seven mutations in the conserved core motifs abrogated the function of Vas with regard to embryonic viability.

Interestingly, eGFP-Vas^Δ636-646^, despite its inability to restore pole cell formation to *vas^1^* embryos, was fully able to restore viability even through to adulthood; thus the *vas^1^; egfp-vas^Δ636-646^* allelic combination produces a robust grandchildless phenotype, when the transgene is expressed at the level of the wild-type transgenic control. As in the other assays, eGFP-Vas^R644A^ was fully functional with respect to restoring viability to *vas^1^* embryos, while eGFP-Vas^Δ655-661^ rescued viability at a substantially lower rate than wild-type (p = 0.00054).

To determine whether these effects on viability correlate with defects in anterior-posterior patterning, we performed *in situ* hybridization for *ftz* mRNA on *vas^1^* embryos expressing the different *egfp-vas* transgenes (Fig. 8B). In wild-type blastoderm-stage embryos *ftz* mRNA is expressed in seven transverse stripes along the anterior-posterior axis, while in *vas^1^* embryos *ftz* mRNA is usually expressed in only four stripes, with loss of stripes 4, 5, and 6 that correspond to future abdominal segments. For all N-terminal deletions within the interval 15-127, the average number of *ftz* stripes was above six and the embryos with seven stripes represented the most frequent class. However, the average number of *ftz* stripes in *vas^Δ3-139^* decreased to 5.69±0.2 (p = 0.038) which was associated with about a 30% decrease in the number of embryos with seven stripes. For Δ3-200 and all mutations in conserved DEAD-box residues, similar to *vas^1^* the average number of *ftz* stripes was less than four, and embryos with four *ftz* stripes were predominant, indicating a failure of these constructs to rescue posterior patterning and thus embryonic viability. Seven *ftz* stripes predominated in embryos expressing each of the constructs mutated at the C-terminal end, except for Δ655-661 where the average was 5.47±0.19 (p = 0.0028) and in approximately 60% of these embryos fewer than seven *ftz* stripes were present.

Taken together, these results indicate that the conserved domains that Vas shares with other DEAD-box helicases are essential for posterior patterning, and that other residues in the intervals between amino acids 140-200 and 655-661 also contribute to this function.

## Discussion

Mutant forms of Vas with abrogated DEAD-box motifs can support oogenesis and can localize to the posterior pole of the stage-10 oocyte, but RNA helicase activity of Vas is required for most of its cellular and developmental functions.

This comprehensive analysis of the *in vivo* activities of a series of eGFP-Vas proteins allows us to draw several conclusions as to how its various functions are related to different motifs within the protein. Surprisingly, we found that most mutant eGFP-Vas proteins carrying non-conservative amino acid changes that would be expected to abrogate fundamental DEAD-box helicase functions such as ATP binding or RNA binding can nevertheless restore fecundity to a *vas*-null mutant. Furthermore, most of these same mutant proteins can localize to the posterior pole of the stage-10 *vas^1^* oocyte. This argues that whatever role Vas performs to allow oogenesis to progress to completion, and also its posterior localization, does not require it to function as an RNA helicase. We speculate that these functions of Vas may be fulfilled through it operating as an inert scaffolding molecule. The exceptional mutations that do not localize or rescue oogenesis (T546A and D554A, respectively) both affect motif V. Consistent with this, an early study mapped *vas^D5^*, an allele with a phenotype of similar severity as *vas^PH165^* and that produces a protein that fails to localize, to a point mutation in motif V (Liang et al., 1994). Perhaps motif V is within an important region for a scaffolding function, or alternatively mutations in this domain produce alterations in protein folding that abrogate the ability of Vas to serve as a scaffold. Notwithstanding the above, however, most functions of Vas require it to be an active RNA helicase, as translational activation of *grk*, posterior patterning, pole cell specification, and repression of transposon-encoded mRNAs are all not supported by any mutant with alterations in the conserved DEAD-box motifs. We observe as well in many conserved DEAD-box residue mutants (and in Δ3-200) that the nuage appears punctate, and does not fully encircle the nurse cell nuclei. *vas* has previously been implicated in nuage formation in electron microscopy studies (Liang et al., 1994).

### A highly acidic Vas-specific C-terminal motif is required for many Vas functions

At the extreme C-terminal end of Vas orthologues throughout the animal kingdom is found a 5–7 amino acid motif that contains several acidic amino acids and a tryptophan residue at the penultimate or ultimate position (Fig. 1D). This motif is not conserved in other closely related DEAD-box proteins. The C-terminal seven amino acids of human DDX3 or *Drosophila* Belle, DEAD-box proteins that are most closely related to Vas, both include two consecutive tryptophan residues but include only one aspartate residue each, thus their C-terminal ends have substantially diverged from Vas and the human Vas orthologue DDX4. The C-terminal ends of *Drosophila* RM62 or human DDX5, the next most closely related DEAD-box proteins to Vas, include neither tryptophan residues nor acidic residues. In this study we show that this Vas-specific C-terminal motif is nearly as essential to its function *in vivo* as the conserved DEAD-box domains. The mutant form of eGFP-Vas deleted for this motif does not support translational activation of *grk*, pole cell specification, and its activity with respect to posterior patterning and repression of transposon-encoded mRNAs is clearly reduced. These results indicate that this C-terminal domain is a key feature of Vas that distinguishes it functionally from other closely related DEAD-box helicases.

We made two other noteworthy observations concerning the C-terminal region of Vas. First, we identified a motif (636-646) that is essential for fine-tuning the distribution of Vas at the posterior pole of the early embryo. When this motif is deleted, posterior localization of Vas is more diffuse, affecting pole cell specification. It is possible that some mechanism prevents diffusion of Vas along the cortex upon its localization to the posterior pole, and that this mechanism depends on the 636-646 motif. Alternatively, there might be an active process to generate a more focused domain of Vas following its initial localization to the posterior region, and the 636-646 motif is essential for engaging Vas with that process. Since Vas^Δ636-646^, when it is expressed at high enough levels, can induce germ cell formation it is not inherently defective for this function. Second, we found that deletion of amino acid 617, previously shown to affect the Vas-eIF5B interaction, also results in a failure to repress transposon-encoded mRNAs. This could suggest that the role of Vas in transposon silencing involves translational regulation, but the accumulation of Vas in the nuage and the direct association of Vas with piRNAs support a more direct role (Xiol et al., 2014; Zhang et al., 2012). We speculate that deletion of amino acid 617 disrupts not only the eIF5B interaction but another interaction as well that is involved with piRNA processing.

### The rapidly-evolving N-terminal region contributes to Vas function

After the first eight or nine amino acids at the extreme N-terminus, which are highly conserved among Vas orthologues in different Drosophilids, there is a rapidly evolving segment of the protein that extends for approximately the next 170 amino acids in *D. melanogaster* (Fig. 1A). Strong sequence conservation among species begins again near the DINNN motif (amino acids 184-188) that binds to two Cullin-RING E3 ligase specificity receptors, Gustavus (Gus) and Fsn (Kugler et al., 2010). We found that eGFP-Vas proteins with deletions within the region 15-110 are able to support all functions of Vas that we examined. Analysis of more extensive deletions, however, reveals several roles for the N-terminal domain. Deletions affecting the region between amino acids 110-139 produce inconsistent effects on transposon mRNA silencing, suggesting that these forms of Vas are close to a threshold level of activity with respect to this function, and that this region of the protein has at least an accessory role in piRNA processing. Rapid evolution of some germ line-expressed genes must contribute to the reproductive isolation of species, and derepression of transposon activity has been observed in *D. melanogaster/D. simulans Hmr/+* hybrid females (Kelleher et al., 2012). It is interesting in this context that a segment of the rapidly evolving region of Vas might be implicated in transposon silencing, as it suggests a potential mechanism underlying hybrid sterility.

Complete deletion of the N-terminal region (Δ3-200) abrogates the ability of eGFP-Vas to support posterior patterning and pole cell specification, while eGFP-Vas^Δ3-139^ supports these functions to some extent. The larger deletion includes the DINNN motif, which may contribute to these functions. However, as *gus* and *Fsn* mutations do not completely abrogate posterior patterning and pole cell specification (Kugler et al., 2010), other motifs probably contribute as well. Our analysis also indicates that the integrity of three RGG motifs in Vas^Δ17-110^ is necessary for its function in pole cell specification.

Our results indicate that different functions of Vas require different domains of the protein, which in turn implies that its different functions involve a diversity of molecular associations. DEAD-box helicases often function as components of ribonucleoprotein particles (RNPs), and it is likely that each mutation in eGFP-Vas which affects particular functions might also affect its ability to assemble into some RNPs but not into others. The eGFP-Vas transgenes that we have generated open the door for future proteomic analysis that could address this issue and link specific molecular associations with cellular and developmental functions.

## Supplementary Material

Supplementary Material
