# Supplementary material for: In vivo mapping of the functional regions of the DEAD-box helicase Vasa
Source: Biol Open. 2015 Mar 20;4(4):450–62. doi: 10.1242/bio.201410579 (PMC4400588; doi:10.1242/bio.201410579)
Supplement: Supplementary Material [file supp_4_4_450__index.html]

In vivo mapping of the functional regions of the DEAD-box helicase Vasa — Supplementary Material 

# *In vivo* mapping of the functional regions of the DEAD-box helicase Vasa

## bio.201410579 Supplementary Material

**Files in this Data Supplement:**

- Supplementary Material - Mehrnoush Dehghani and Paul Lasko doi: 10.1242/bio.201410579
- Movie 1 - **Germ cell formation in *vas1; egfp-vas+* embryos.** Posterior nuclei divide asynchronously from somatic cells and develop into eGFP-Vas positive germ cells.
- Movie 2 - **Germ cell formation in *vas1; egfp-vasΔ636-646* embryos.** Pole buds in *vas1; egfp-vasΔ636-646* fail to develop into germ cells. Nuclear divisions at the posterior germ cell region of *vas1; egfp-vasΔ636-646* embryos remain synchronous with the somatic nuclei.
