# Supplementary material for: In vivo mapping of the functional regions of the DEAD-box helicase Vasa
Source: Biol Open. 2015 Mar 20;4(4):450–62. doi: 10.1242/bio.201410579 (PMC4400588; doi:10.1242/bio.201410579)
Supplement: Supplementary Material [file supp_bio.201410579_bio.201410579-s1.pdf]

Biology Open (2015) 000, 1–13 doi:10.1242/bio.201410579

Biology Open

Supplementary Material

Mehrnoush Dehghani and Paul Lasko doi: 10.1242/bio.201410579

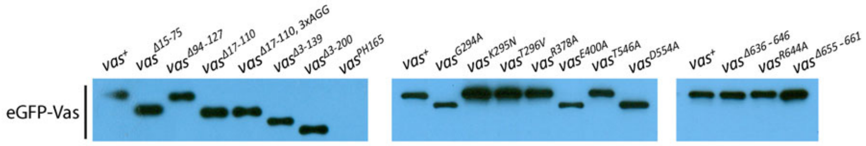

Fig. S1. The same western blot as in Fig. 2B stained with anti-GFP to compare expression levels of eGFP-Vas proteins in ovaries from vas^1/+ females carrying the different constructs.

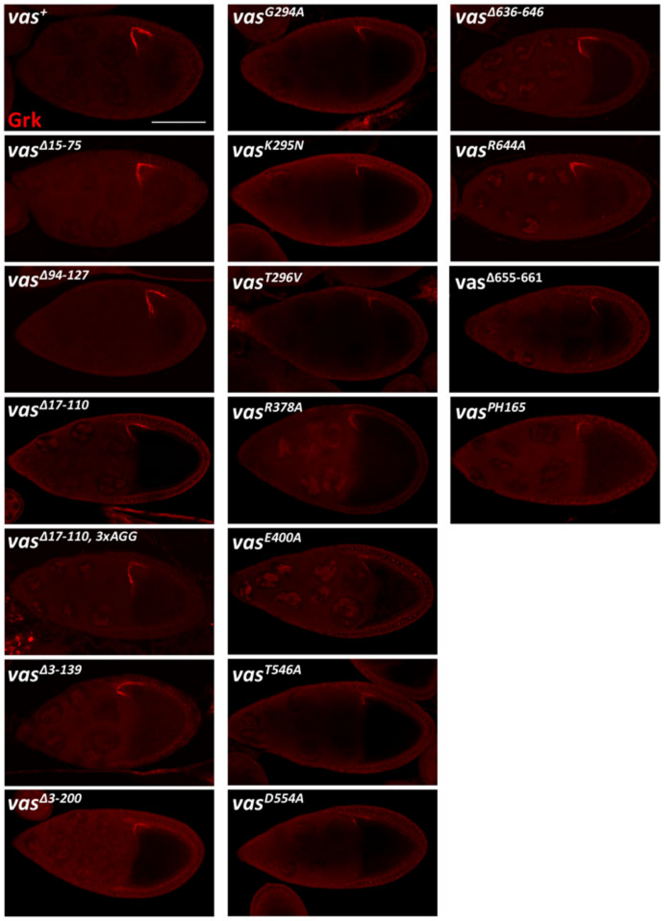

Fig. S2. Representative confocal images of Grk immunostaining in the subset of stage 8 egg chambers that were positively stained in each genotype. Scale bar=50 µm.

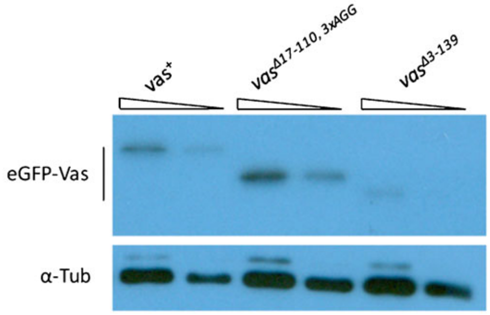

Fig. S3. A comparison between eGFP-Vas^{+}, eGFP-Vas$^{\Delta17\text{-}110,\ 3xAGG}$ and eGFP-Vas$^{\Delta3\text{-}139}$ for their stability in 0-2 h embryos from vas^1/+ females. Top panel; western blot using a GFP antibody. Bottom panel: the same blot was stained for α-tubulin as a loading control. In the rightmost lane of each pair half as much lysate was loaded. The low level of eGFP-Vas$^{\Delta3\text{-}139}$ in embryos suggests that this protein may be unstable.

Biology Open (2015) 000, 1–13 doi:10.1242/bio.201410579

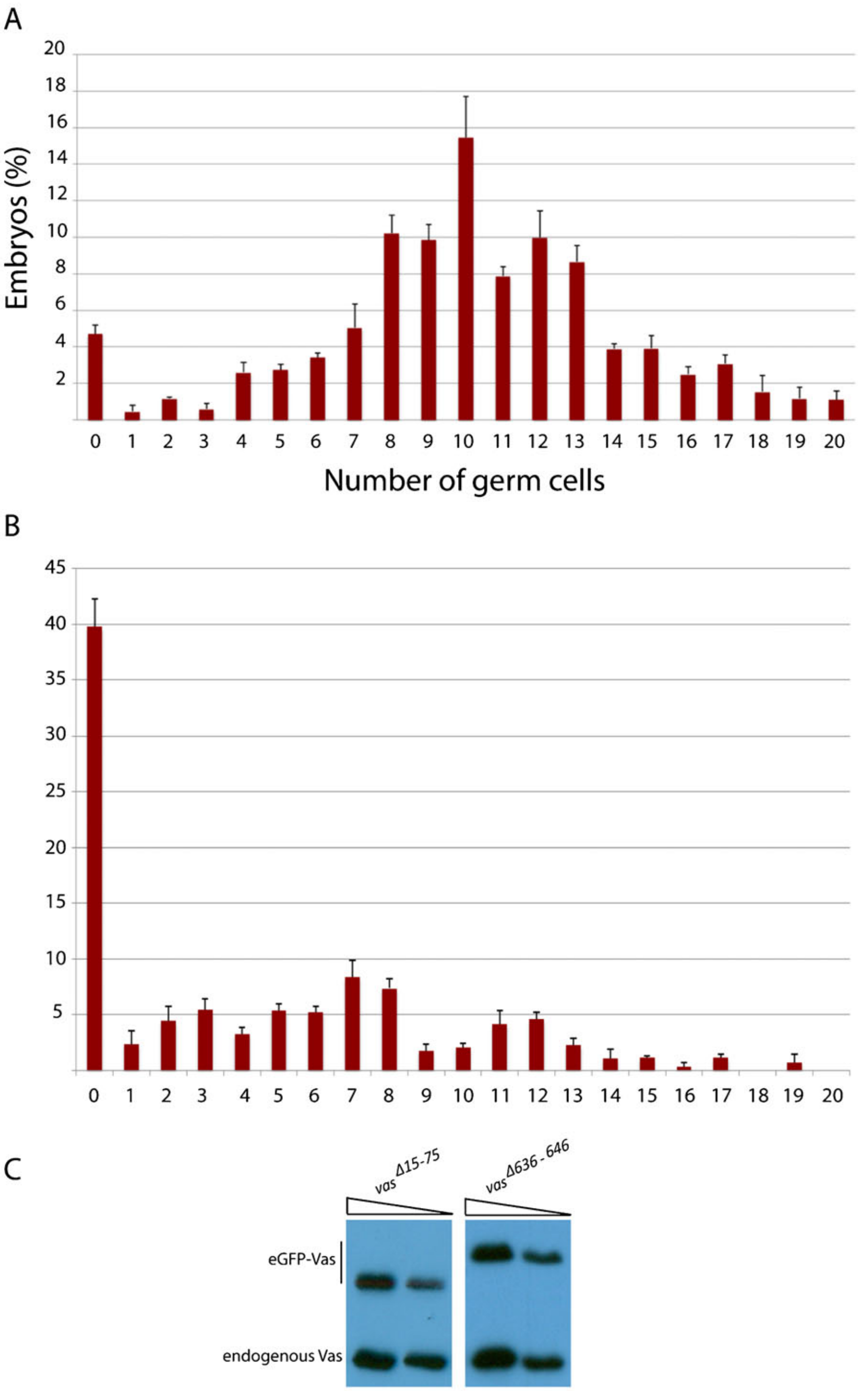

Fig. S4. ***vas***^{1}***; egfp-vas***$^{\Delta 15\text{-}75}$ **and** ***vas***^{1}***; egfp-vas***$^{\Delta 636\text{-}646}$ **are compared for their variability in the number of pole cells per stage 4-5 embryo.** Pole cell numbers have a normal distribution in *vas*^{1}*; egfp-vas*$^{\Delta 15\text{-}75}$ embryos (A), whereas in a *vas*^{1}*; egfp-vas*$^{\Delta 636\text{-}646}$ line (B) that has a similar expression level of eGFP-Vas, the number of pole cells in embryos is highly variable with a bias toward lower values. Error bars represent SEM from three replicates with more than 50 embryos each. (C) Western blots from 0-2 h embryos compare expression levels of eGFP-Vas in *egfp-vas*$^{\Delta 15\text{-}75}$ and egfp-*vas*$^{\Delta 636\text{-}646}$ lines. In the rightmost lane of each pair half as much lysate was loaded. A Vas antibody was used to detect both the eGFP-Vas and the endogenous Vas.

Biology Open (2015) 000, 1–13 doi:10.1242/bio.201410579

Table S1. Results of the statistical analyses

	vas^{+}	$vas^{\Delta15-75}$	$vas^{\Delta94-127}$	$vas^{\Delta17-110}$	$vas^{\Delta17-110,\ 3xAGG}$	$vas^{\Delta3-139}$	$vas^{\Delta3-200}$	vas^{G294A}	vas^{K295N}
Number of eggs produced/female[1]	1.05E-07	1.22E-05	1.13E-11	1.43E-07	1.64E-07	9.71E-09	8.46E-10	6.44E-08	1.64E-07
Embryos with two dorsal appendages (%)[2]	—	0.0910	7.78E-08	5.91E-08	0.0047	0.0001	1.50E-12	1.07E-20	6.64E-10
Stage 8 egg chambers with Grk crescent (%)[2]	—	0.0473	0.8568	0.4981	0.9018	0.2094	0.0291	0.0037	0.0025
HeT-A overexpression[2]	—	0.3878	0.2320	0.5582	0.4822	0.1823	0.0039	0.0402	0.0237
Embryos with germ cell (%)[2]	—	0.0262	0.5819	0.7785	0.0221	0.0181	0.0128	0.0128	0.0128
Embryonic viability (%)[2]	—	0.0390	0.6891	0.5599	0.8610	0.0443	1.66E-05	1.52E-05	1.52E-05
Average number of stripes/embryo[2]	—	0.1402	0.2807	0.5552	0.8248	0.0376	9.33E-18	2.29E-23	7.01E-20

	vas^{T296V}	vas^{R378A}	vas^{E400A}	vas^{T546A}	vas^{D554A}	$vas^{\Delta617}$	$vas^{\Delta636-646}$	vas^{R644A}	$vas^{\Delta655-661}$	vas^{PH165} or vas^{1}	$vas^{PH165}/Df(2L)A267$
Number of eggs produced/female[1]	0.0002	1.86E-06	0.0001	6.32E-08	0.0846	—	9.28E-08	1.04E-09	3.95E-10	—	0.8269
Embryos with two dorsal appendages (%)[2]	3.24E-20	1.48E-21	1.71E-17	1.70E-20	4.29E-15	—	2.81E-06	4.13E-15	8.63E-19	3.07E-15	—
Stage 8 egg chambers with Grk crescent (%)[2]	0.0043	0.0009	0.0045	0.0041	0.0043	—	0.7973	0.2671	0.0018	0.0037	—
HeT-A overexpression[2]	—	—	—	—	0.0101	0.0024	0.7551	—	0.1422	0.0216	0.0239
Embryos with germ cell (%)[2]	0.0128	0.0128	0.0128	0.0128	0.0128	—	0.0130	0.8308	0.0131	0.0128	—
Embryonic viability (%)[2]	1.52E-05	1.52E-05	1.52E-05	1.52E-05	1.52E-05		0.1930	0.6114	0.0005	1.52E-05	—
Average number of stripes/embryo[2]	3.28E-19	3.20E-18	1.89E-19	1.05E-19	2.13E-19	—	0.7425	0.2970	0.0028	7.62E-22	—

The p-values calculated in each assay are listed. Different genotypes were compared to vas^{PH165} for egg-laying assays, and to *egfp-vas*$^{+}$ for all the other assays.

1: compared to vas^{PH165}

2: compared to vas^{+}

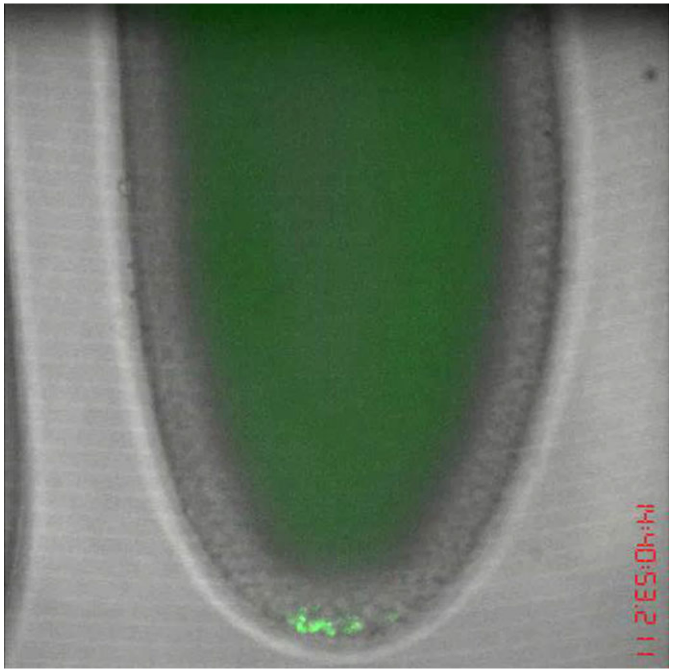

Movie 1. Germ cell formation in *vas*^{1}*; egfp-vas*$^{+}$ embryos. Posterior nuclei divide asynchronously from somatic cells and develop into eGFP-Vas positive germ cells.

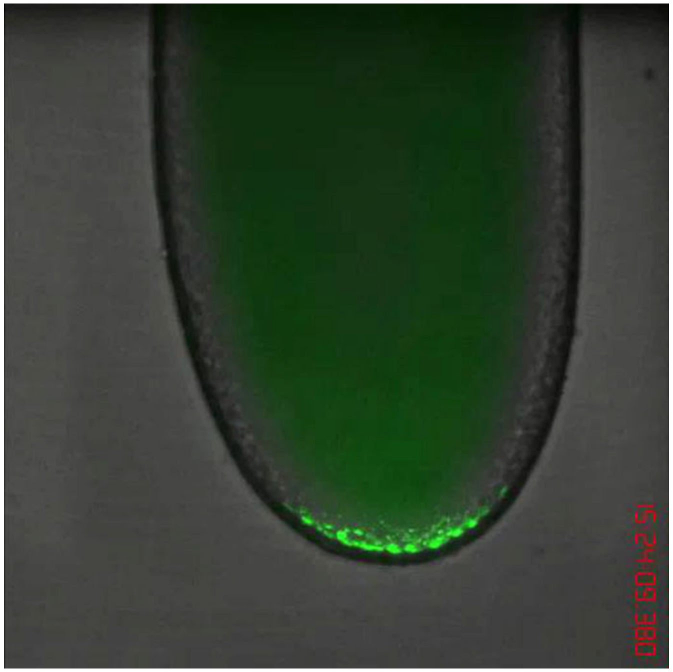

Movie 2. Germ cell formation in *vas*^{1}*; egfp-vas*$^{\Delta636-646}$ embryos. Pole buds in *vas*^{1}*; egfp-vas*$^{\Delta636-646}$ fail to develop into germ cells. Nuclear divisions at the posterior germ cell region of *vas*^{1}*; egfp-vas*$^{\Delta636-646}$ embryos remain synchronous with the somatic nuclei.
